# Endocan, a novel glycoprotein with multiple biological activities, may play important roles in neurological diseases

**DOI:** 10.3389/fnagi.2024.1438367

**Published:** 2024-09-12

**Authors:** Shuo Liu, Tao Bai, Juan Feng

**Affiliations:** ^1^The Fourth People’s Hospital of Shenyang, Shenyang, Liaoning, China; ^2^Department of Neurology, Shengjing Hospital of China Medical University, China Medical University, Shenyang, China

**Keywords:** angiogenesis, brain, endocan, endothelial cell, inflammation, neurological disease

## Abstract

Endothelial cell specific-1 (*ESM-1*), also known as endocan, is a soluble dermatan sulfate proteoglycan that is mainly secreted by endothelial cells. Endocan is associated with tumorigenesis and cancer progression and is also related to cardiovascular disorders, autoimmune diseases, and sepsis. The phenylalanine-rich region and linear polysaccharide of endocan are necessary for the protein to exert its biological functions. Elevated plasma endocan levels reflect endothelial activation and dysfunction. In addition, endocan participates in complex inflammatory responses and proliferative processes. Here, we reviewed current research on endocan, elaborated the protein’s structure and biological functions, and speculated on its possible clinical value in nervous system diseases. We conclude that endocan may be a glycoprotein that plays an important role in neurological disorders.

## 1 Introduction

Endocan is a novel dermatan sulfate (DS) proteoglycan (PG) that is specifically expressed in activated endothelial cells. Unlike other glycoproteins, endocan exists mainly in the free form in serum (Bechard et al., 2000). To date, it has been verified that serum endocan levels are associated with various malignancies, systemic inflammation, vascular disorders, and use of certain medications (Entezarian et al., 2022; Faulkner et al., 2018; Ghods et al., 2014; Reyes et al., 2016; Chen et al., 2021). In addition, endocan molecules participate in a wide range of pathophysiological processes, such as inflammatory responses, angiogenesis, vascular permeability, and tumor metastasis via interacting with cytokines or inflammatory mediators.

Inflammation and vascular dysfunction are essential pathological changes in neurological disorders. However, there is limited evidence regarding the roles of endocan in nervous system diseases, and it is not known whether this molecule is significant in neurological diseases. In this article, we review the structure and biological importance of endocan and discuss its potential roles in neurological disorders, which may facilitate new insights in this field.

## 2 Structure and regulation of endocan

### 2.1 The structure of endocan

Endocan was initially named endothelial special molecule-1(*ESM-1*) because of its specific expression in endothelial cells. Due to its glycoprotein characteristics, it was later named endocan in 2001 (Beìchard et al., 2001a). With in-depth studies, it has been confirmed that endocan expression is not restricted to endothelial cells. Tissues and cells with proliferative activity are the main source of secreted endocan in serum. Glandular tissues, the epithelial cells of kidneys and lungs, and cardiac muscle cells also express endocan. It is not expressed in relatively resting tissues or cells, such as the endothelium of the large main arteries and the spleen (Zhang et al., 2012).

With a molecular weight of 50kDa, endocan is a soluble DSPG that exists in a free state in the circulation (Bechard et al., 2000). Endocan regulates several cellular processes, such as cell adhesion, migration, and proliferation (Lassalle et al., 1996). Unlike most PGs located in the extracellular matrix or related to cell membranes, the soluble nature of endocan allows it to participate in systemic physiological processes.

**FIGURE 1:**
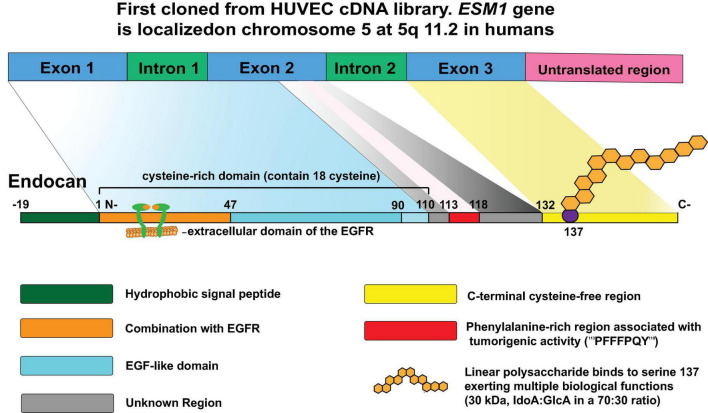
The structure of endocan: Endocan was first cloned from a human umbilical vein endothelial cell (HUVEC) cDNA library in 1996. Located on chromosome 5 at position 5q11.2, ESM1 consists of three exons, two introns, and a 3′ untranslated region. Exon 1 and a part of exon 2 encode the N-terminal cysteine-rich region containing 110 amino acids; the rest of exon 2 encodes residues 111–132 containing functional region that is rich in phenylalanine; and exon 3 encodes the C-terminal region composed of 33 amino acid residues that contain the proteoglycan binding site at serine 137. A mature endocan molecule is composed of a polypeptide of 165 amino acids (20kDa) and a single dextran sulfate chain that includes 32 disaccharide residues (30kDa), which has several functional regions allowing the protein to participate in various biological processes.

In 1996, endocan was first cloned from a human umbilical vein endothelial cell (HUVEC) cDNA library (Lassalle et al., 1996). Endocan is encoded by ESM1 (Lassalle et al., 1996), which is localized on chromosome 5 at position 5q11.2 in humans (Figure 1). ESM1 consists of three exons, two introns, and a 3′ untranslated region. Exon 1 and a part of exon 2 encode the N-terminal cysteine-rich region containing 110 amino acids, exon 2 encodes the phenylalanine-rich functional region, and exon 3 encodes the C-terminal region that is composed of 33 amino acid residues that contain the PG-binding site at serine 137 (Figure 1).

**FIGURE 2:**
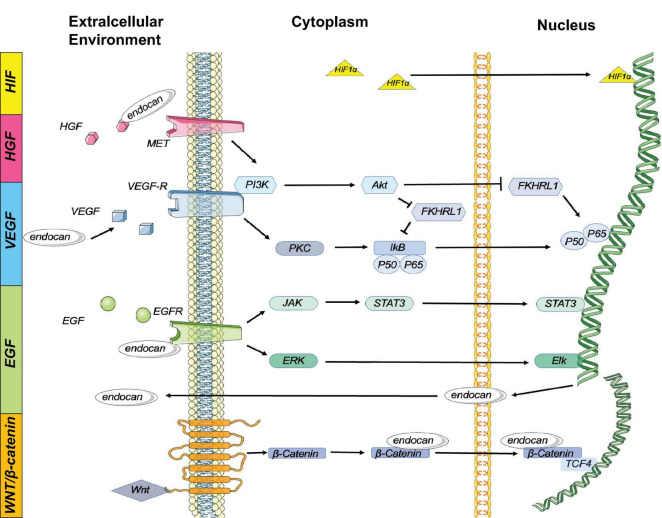
Endocan is involved in several signaling pathway: Expression of endocan is regulated by multiple cytokines. In turn, endocan can influence several biological reactions:1. HIF-1α Pathway: HIF-1α can induce the expression of endocan. 2. HGF/MET Pathway: Endocan binds to and activates HGF to stimulate MET signaling. The HGF/MET signaling pathway works synergistically with VEGF/VEGFR to promote angiogenesis and tumor growth. 3. VEGF Pathway: Endocan enhances VEGF bioactivity, and VEGF induces endocan gene expression by activating the PKC/NF-κB signaling pathway. Additionally, VEGF stimulates the PI3K/Akt signaling pathway, which inhibits the endocan expression induced by FKHRL1. 4.EGFR Pathway: The activation of EGFR induces endocan expression via the JAK/STAT3 and ERK/Elk cascades. 5. Wnt/β-Catenin Pathway: Nuclear endocan interacts with β-catenin, retaining β-catenin in the nucleus by stabilizing its interaction with TCF4 and stimulating the transcriptional activation of Wnt/β-catenin signaling targets. Additionally, activation of Wnt/β-catenin signaling promotes nuclear translocation of endocan.

Mature endocan is composed of a polypeptide of 165 amino acids (20kDa) and a single DS chain that includes 32 disaccharide residues (30kDa) (Figure 1). According to the structure characteristics and functions, the polypeptide chain is divided into several regions (Figure 1). The first 19 residues form a functional NH2-terminal hydrophobic signal sequence for extracellular secretion of this protein and which is removed after maturation (Lassalle et al., 1996). The amino acid region from 1 to 110 was identified as cysteine-rich domain, which contains 18 cysteine residues. Moreover, the region comprising amino acids 1-46 was further identified as being associated with the extracellular domain of the epidermal growth factor receptor (EGFR) and is involved in tumorigenesis (Yang et al., 2020). In prostate cells, this region was found to interact with β-catenin (Pan et al., 2021). This interaction retains β-catenin in the nucleus by stabilizing its interaction with T-cell factor complex (TCF4) and stimulates the transcriptional activation of Wnt/β-catenin signaling targets. In addition, activation of Wnt/β-catenin signaling promotes nuclear translocation of endocan (Pan et al., 2021; Figure 2). The remaining non-cysteine amino acid sequence (containing 55 amino acids) is divided into three functional regions: the endothelial growth factor-like region, phenylalanine-rich region, and C-terminal region (Delehedde et al., 2013). The phenylalanine-rich region plays an important role in promoting proliferation, while the roles of the endothelial growth factor-like region of endocan remain inconclusive. In addition, the core protein is covalently linked to a glycosaminoglycan type linear polysaccharide chain via serine 137 (Lassalle et al., 1996). The linear polysaccharide contains N-acetyl-galactosamine and uronic acid (iduronic acid [IdoA]: glucuronic acid [GlcA] in a 70:30 ratio). IdoA is important for endocan to exert regulatory function. Mouse polypeptide endocan shares 75% homology with that of humans and 95% homology with that of rats (Depontieu et al., 2012). Considering that the domain structure is the same across mouse, rat, and human polypeptides, it is possible to explore the biological function of endocan under different physiological and pathological conditions.

ESM1 encodes two different products. One is a mature endocan molecule, while the other lacks the exon 2 region (named endocan△2). The absence of exon 2 was suggested to impair the glycosylation of endocan and induce protein oligomerization (Depontieu et al., 2008). Endocan without a normal phenylalanine-rich region or glycan moiety has been reported to lack a cell proliferation role (Aitkenhead et al., 2002).

With a half-life of 1 hour, endocan is biodegraded mostly by cathepsin G (Madhivathanan et al., 2016). By neutrophil-secreted cathepsin G-mediated hydrolysis, endocan can be broken down to 1–111, 1–115, or 1–116 endocan peptide fragments, lacking glycosylation, which are collectively called P14 (14kDa) endocan (De Freitas Caires et al., 2013). The P14 endocan degradation product exhibits a rigid structure, probably because of the high number of disulfide bonds (De Freitas Caires et al., 2013). Given their low molecular weight, P14 endocan fragments are easily removed by the renal glomerulus. Studies have confirmed that serum P14 endocan levels are positively associated with the chronic kidney disease stage and are negatively related to the glomerular filtration rate (Nalewajska et al., 2020).

### 2.2 Regulation of endocan

#### 2.2.1 Growth factor signaling pathways

Endocan gene expression is influenced by several growth factors such as VEGF-A, VEGF-C, HGF/MET, FGF-2, and EGFR, involving complex signaling mechanisms including PKC/NF-κB, PI3K/Akt, and JAK/STAT3. These growth factors interact with and enhance each other’s bioactivity, contributing to angiogenesis and tumor growth (Abid et al., 2006). For example, VEGF-A and VEGF-C induce endocan expression by activating VEGFR, which involves the PKC/NF-κB and PI3K/Akt pathways (Abid et al., 2006; Figure 2). HGF/MET signaling synergizes with VEGF/VEGFR to promote angiogenesis and tumor growth, while EGFR activation induces endocan expression through the JAK/STAT3 and ERK/Elk cascades (Yang et al., 2020; Figure 2). These interactions highlight endocan’s multifaceted role in growth factor signaling.

#### 2.2.2 Proinflammatory cytokines and bacterial lipopolysaccharide (LPS)

Proinflammatory cytokines, such as TNF-αα and IL-1β, and bacterial LPS significantly upregulate endocan expression by activating inflammatory signaling pathways, including NF-κB (Lo Gullo et al., 2021). Endocan, in turn, can induce the expression of inflammatory cytokines and activate proinflammatory signaling pathways (Lo Gullo et al., 2021).

#### 2.2.3 Epigenetic and other regulatory factors

Epigenetic modifications, such as promoter demethylation, and other factors like HIF-1α, IFN-γ, and IL-4 also modulate endocan expression (Lee H. G. et al., 2014). For instance, under hypoxic conditions, HIF-1α upregulates endocan expression by promoting adhesion between monocytes and endothelial cells (Sun H. et al., 2019; Figure 2). IFN-γ and IL-4 appear to have negative effects on endocan release (Rennel et al., 2007; Nirala et al., 2015).

#### 2.2.4 Summary and future directions

Endocan expression is regulated by various factors, including proinflammatory cytokines, bacterial LPS, growth factors, and hypoxic conditions. Each regulatory pathway has unique advantages and disadvantages. For example, VEGF-induced endocan expression is crucial for angiogenesis but may lead to pathological vascular permeability, whereas hypoxia-induced expression is essential for tissue response to low oxygen levels but can exacerbate inflammation. Future research should focus on comparing these regulatory pathways in different disease contexts to better understand their specific roles and therapeutic potential.

## 3 Endocan participates in pathophysiological processes

### 3.1 Endocan in inflammation

**FIGURE 3:**
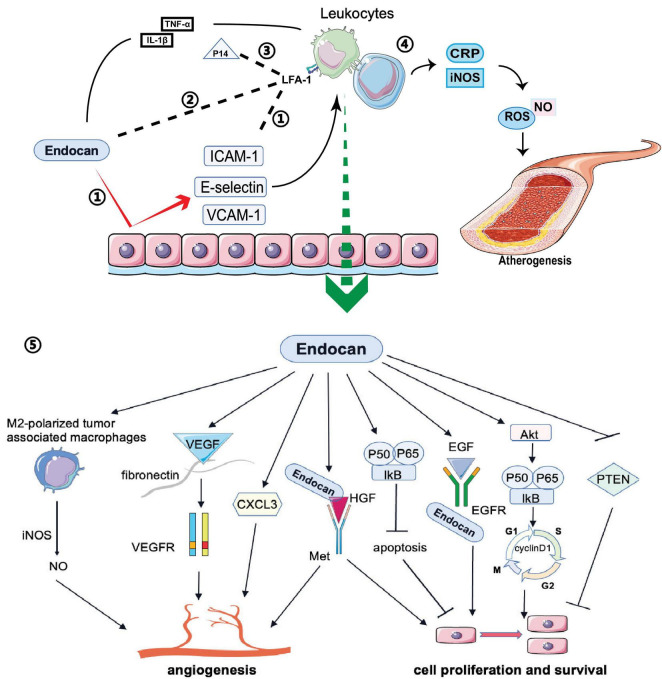
Biological functions of endocan: Endocan and P14 participate in various biological processes: 1) Endocan promotes the release of E-selectin ICAM-1 and VCAM-1 from endothelium, which induces the recruitment and adhesion of circulating leukocytes. 2) Endocan can directly bind to LFA-1, inhibiting leukocyte recruitment and extravasation by blocking LFA-1 and ICAM-1 interactions. 3) P14 endocan can restore the interaction between ICAM-1 and LFA-1 by competing with endocan for binding to LFA-1. 4) Endocan promotes the production and release of NO and ROS from macrophages, which contribute to the atherogenesis. 5) Endocan participates in angiogenesis, cell proliferation, and survival via several pathways.

Endocan expression can be induced by various proinflammatory cytokines (Cakmak et al., 2016; Kumar and Mani, 2024). For example, TNF-α secreted by monocytes or macrophages increases endocan expression, promoting inflammatory reactions. Additionally, IL-1β activates the NF-kB pathway to stimulate endocan expression (Scuruchi et al., 2021; Scuruchi et al., 2023; Figure 3). In vitro studies have shown that elevated endocan levels further promote inflammatory responses in macrophages by inducing the expression of iNOS and C-reactive protein (CRP), and increasing the production of nitric oxide (NO) and reactive oxygen species (ROS), which contribute to atherogenesis (Kartik Kumar et al., 2020; Figure 3). Endocan also promotes the release of E-selectin, ICAM-1 and VCAM-1 from inflamed endothelial cells, aiding the recruitment and adhesion of circulating leukocytes to the vascular endothelium (Lee W. et al., 2014; Sun H. et al., 2019; Figure 3). This interaction may be a crucial initial event in angiogenesis, atherosclerosis, and vascular inflammation (Chen et al., 2021). Moreover, endocan may influence the levels of soluble forms of these cell adhesion molecules (sCAMs) by regulating the expression of their membrane-bound counterparts (Tayman et al., 2020). sCAMs can serve as biomarkers of endothelial activation, reflecting changes in the expression of CAMs on the surface of endothelial cells (Pawlak et al., 2015).

Conversely, endocan may have anti-inflammatory effects in certain contexts. Recent studies suggest that in acute lung inflammation, endocan can bind directly to integrin CD11a/CD18 (LFA-1), inhibiting leukocyte recruitment and extravasation by blocking LFA-1 and ICAM-1 interactions (Beìchard et al., 2001b; Gaudet et al., 2019; Figure 3). Interestingly, the P14 fragment of endocan, which is cleaved by the neutrophil-derived protease cathepsin G, competes with full-length endocan for LFA-1 binding, restoring the ICAM-1 and LFA-1 interaction and facilitating leukocyte extravasation (Kali and Shetty, 2014; Gaudet et al., 2020; Figure 3). This fragment is found at elevated levels in the plasma of sepsis patients, indicating it may be a marker of neutrophil activation in inflammatory conditions (Kali and Shetty, 2014). Zhang et al. (2018) demonstrated that in an acute lung injury mouse model induced by LPS, endocan inhibited LPS-induced apoptosis of pulmonary epithelial cells and reduced inflammation by lowering cytokine levels, including TNF-α, INF-γ, IL-1β, and IL-6. The LFA-1/ICAM-1 pathway is pivotal in respiratory inflammation, and endocan levels are lower in patients with acute lung injury and acute respiratory distress syndrome (Kechagia et al., 2016). In chronic kidney disease patients with cardiovascular disease, increased endocan levels were associated with a lower percentage of circulating lymphocytes, potentially due to increased adherence of lymphocytes to the endothelium (Samouilidou et al., 2022). Therefore, while endocan seems to have an anti-inflammatory effect by blocking leukocyte migration, its levels are elevated in inflammatory conditions and may reflect the activation state of neutrophils, lymphocytes, and the endothelium. More research is needed on how endocan and its fragments directly impact circulating leukocyte numbers and function.

Furthermore, knockdown of ESM1, the gene encoding endocan, increases the expression of the NF-kB inhibitor IkBα and decreases the expression of phosphorylated IkBα, phosphorylated-P65, and nuclear P65, indicating deactivation of the NF-kB signaling pathway (Sun L. et al., 2019). Activation of the NF-kB pathway by endocan has been shown to promote cell proliferation and survival while suppressing apoptosis (Kang et al., 2012; Sun L. et al., 2019).

### 3.2 Endocan in endothelial dysfunction

Impairment of endothelium-dependent vasodilation, increased endothelial permeability, and glycocalyx degradation are important pathophysiological components of endothelial dysfunction (Bar et al., 2019). Currently, several techniques are used to assess NO-dependent vasodilation, which enables the diagnosis of endothelial malfunction in clinical studies (Flammer et al., 2012). In clinical practice, flow-mediated dilation in the brachial artery and reactive hyperemia in the peripheral circulation are two major diagnostic parameters measured by ultrasonography and tonometry of the finger, respectively (Bonetti et al., 2004; Frolow et al., 2015). Higher endocan levels were found in patients with higher arterial pulse wave velocity, which may reflect the degree of arterial stiffness (Poon et al., 2019).

It has been suggested that glycocalyx, composed of PGs and glycoproteins, is an essential component of the vascular endothelial surface layer, which covers the luminal surface of the endothelium and plays important roles in the pathophysiological process of leukocyte-endothelium interactions, thrombosis, vascular permeability alterations, and vascular inflammation (Sieve et al., 2018). Research has found that knocking down endocan significantly downregulates the expression of angiogenesis-associated genes in IL-1β-activated chondrocytes, including VEGF-A, MMP-9, MMP-13, and VEGFR-2. This indicates that endocan plays a key regulatory role in angiogenesis and inflammatory responses (Scuruchi et al., 2023).

Recent studies suggest that endocan can directly increase endothelial permeability and oxidative stress. Endocan stimulates endothelial cells to secrete proinflammatory cytokines and increases the production of reactive oxygen species (ROS), leading to oxidative stress and endothelial dysfunction. Additionally, endocan alters nitric oxide (NO) production by inhibiting the AKT/eNOS pathway and activating the NF-κB/iNOS pathway, contributing to endothelial barrier disruption and vascular inflammation (Kumar and Mani, 2021).

Glycocalyx deterioration is suggested to be one of the most important manifestations of endothelial dysfunction (Bar et al., 2019). Endocan, as a biomarker of glycocalyx disruption, might be helpful in the diagnosis and treatment of various vascular disorders, such as atherosclerosis. Endothelial injury may be an incipient lesion in this chronic, progressive inflammatory process. Deterioration of the glycocalyx results in increased endothelial permeability and endothelial barrier disruption (Becker et al., 2010; Lukasz et al., 2017), which could also induce a proinflammatory endothelial response (Voyvodic et al., 2014). A study confirmed that glycocalyx disruption is progressive and may be a reliable marker of endothelial dysfunction (Mitra et al., 2017). As a specific plasma biomarker of glycocalyx disruption, endocan may significantly predict progression and response to treatment (Lin et al., 2023).

### 3.3 Endocan in angiogenesis and cell proliferation

Endocan stimulates mitogenic and migratory processes induced by pro-angiogenic growth factors in endothelial cells (Shin et al., 2008). The angiogenic effects of endocan may depend on VEGF expression. By competing with VEGF-A for fibronectin binding, endocan increases VEGF-A bioavailability, thus promoting the VEGF signaling pathway to regulate vascular outgrowth (Rocha et al., 2014; Figure 3). VEGF can also induce endocan expression (Sarrazin et al., 2006; Rennel et al., 2007; Yazici et al., 2023; Figure 2). Additionally, endocan influences the expression of angiogenic chemokines. For instance, Rebollo et al. (2017) found that endocan affected the expression of C-X-C motif chemokine 3 (CXCL3). This chemokine interacts with its receptors on the surface of tumor cells, activating signaling pathways that lead to cytoskeletal reorganization and thus increased motility. Furthermore, CXCL3 can create a favorable microenvironment for tumor cell invasion by recruiting inflammatory cells and modifying the extracellular matrix (ECM), promoting cell migration and tumor metastasis (Rebollo et al., 2017; Figure 3). Endocan, combined with HGF/SF, can activate the HGF/Met signaling pathway to promote angiogenesis and cell proliferation (Beìchard et al., 2001a; Pan et al., 2022; Figure 3). Moreover, endocan may induce an inflammatory response in macrophages by upregulating iNOS expression, leading to iNOS-mediated NO production in M2-polarized tumor-associated macrophages, which can promote angiogenesis and tumor progression (Kartik Kumar et al., 2020; Perrotta et al., 2018; Figure 3).

Accumulating evidence suggests that endocan participates in cell proliferation. Overexpression of endocan has been confirmed in many types of cancer, with dysregulation observed in both the tumor vasculature and cancer cells (Aliquò et al., 2023). Increased endocan levels are associated with tumor aggressiveness and poor outcomes (Huang et al., 2016; Entezarian et al., 2022). Endocan-promoted tumor cell growth depends on glycosylation and the integrity of the protein core, particularly residues 115-116 in the phenylalanine-rich region (De Freitas Caires et al., 2018; Figure 1). Mechanistic studies have shown that activation of the Akt-dependent NF-kB/cyclin D1 pathway is crucial for endocan-mediated cell-cycle progression from the G1 to S phase, benefiting the proliferation of cancerous cells (Pan et al., 2022; Figure 3). By directly binding to the extracellular region of EGFR, endocan facilitates EGF binding to its receptor, positively impacting tumorigenesis (Yang et al., 2020; Figure 3). PTEN, a cell cycle inhibitor, can reduce cyclin D1 expression during the cell cycle. Silencing endocan has been shown to increase PTEN expression, inhibiting cell growth and metastatic processes (Kang et al., 2011; Figure 3).

## 4 Endocan in nervous system diseases

**FIGURE 4:**
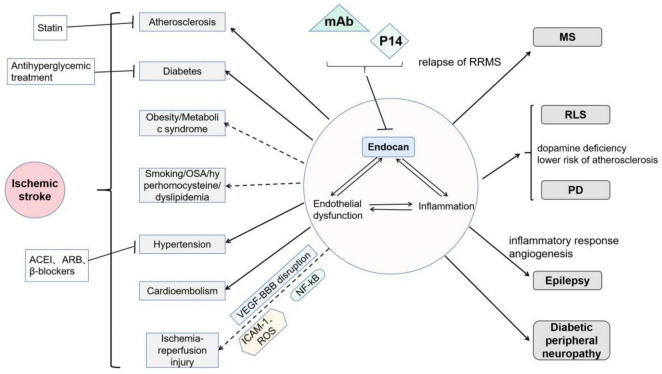
Roles of endocan in neurological disorders: Endocan is involved in endothelial dysfunction and inflammation, and thereby participates in various nervous system diseases: It has been demonstrated that endocan is related to atherosclerosis, diabetes, and hypertension. Moreover, endocan may be related to cardioembolism. Endocan levels may be associated with smoking, obstructive sleep apnea, and hyperhomocysteinemia, but its roles in obesity remain controversial. Endocan may further play important roles in cerebral ischemia-reperfusion injury via VEGF-induced vascular permeability, ICAM-1-mediated leukocyte infiltration, and NF-kB-associated inflammatory response. Endocan has been found to be associated with MS, RLS, PD, epilepsy, and diabetic peripheral neuropathy. Treatments, including statins, antihyperglycemic therapies, and several types of blood pressure drugs, have been shown to decrease serum endocan levels. Moreover, monoclonal antibodies (mAb, as neutralizer) and P14 (as competitive inhibitor) may be potential interventions targeting endocan.

Intricate nerve fibers perform complex brain functions. Neuroglia, nutrient or draining vessels, and several immune cells maintain a constant internal environment in the brain. Endocan expression has been detected in neurons as well as in vascular endothelial cells (Zhang et al., 2012). This DSPG participates in various pathological processes, and its role in systemic disorders may contribute to nervous system impairment. Thus, it is important to uncover the relationship between endocan and nervous system diseases (Figure 4).

### 4.1 Cerebrovascular disease

It has been shown that both endocan and the P14 endocan fragment can be detected in serum of healthy individuals. In the brain, endocan is expressed in neurons but not in neuroglia (Zhang et al., 2012). To date, increasing number of studies have confirmed that endocan is associated with cardiovascular disease (Chen et al., 2021). Endothelial dysfunction and inflammatory processes may explain this correlation, which also play pivotal roles in the pathological course of atherosclerosis, hypertension, diabetes mellitus, etc. Under the influence of these vascular risk factors, patients are at a high risk of cerebrovascular events. Surprisingly, only a few studies have been conducted on this topic. Two studies have demonstrated a correlation between endocan and ischemic stroke (He et al., 2018; Han et al., 2022). It has been suggested that higher endocan levels might be a predictor of poor prognosis by 3 months after an ischemic attack (Han et al., 2022). In patients with silent brain infarction (hyperintense lesion ≥ 3mm in 1 dimension on FLAIR sequence in magnetic resonance imaging, in a patient with no clinical symptoms or with symptoms that were inconsistent with the brain lesion location), higher serum endocan levels were detected (*P* = 0.036) (Erdal et al., 2021).

Below, we discuss the correlation between endocan and cerebrovascular disorders from different perspectives, with a particular focus on ischemic stroke.

#### 4.1.1 Atherosclerosis

Atherosclerosis is a highly prevalent cause of ischemic stroke. The progression and instability of plaques are considered to be the most important mechanisms for ischemic attacks.

Atherosclerosis begins with endothelial dysfunction. In addition, endothelial dysfunction has been detected before the occurrence of well-defined atherosclerosis plaques (Flammer et al., 2012). Circulating monocytes and low-density lipoproteins (LDLs) pass through the impaired endothelium and are deposited in the subendothelial space. Then, the LDLs are oxidized and phagocytized by macrophages to form foam cells, leading to atherosclerotic plaque formation. In addition, increased cytokine production, adhesion molecule expression, and endothelial permeability accelerate this vascular pathological changes (Libby, 2012). Cell adhesion molecules also play essential roles in atherogenesis. The ICAM-1 level remains high in all stages of atherosclerosis, while increased VCAM-1 may reflect unstable plaques. The increased endocan levels, upregulated by the HIF-1α/VEGF pathway enhance the expression of ICAM-1 and VCAM-1 under intermittent hypoxia (Sun H. et al., 2019), which greatly harms the vascular endothelium.

Measurement of carotid intima-media thickness (cIMT) via ultrasonography is a highly sensitive method for monitoring early atherosclerotic changes. Currently, increased cIMT is a reliable indicator of atherosclerosis and cardiovascular disorders (Cao et al., 2022). As mentioned above, endocan participates in the course of atherosclerosis by inducing inflammation in mononuclear phagocytes and endothelial injury. Several studies have found that endocan serum levels are significantly positively correlated with carotid intima-media thickness (Balta S. et al., 2014; Tuzcu et al., 2019; Hassan et al., 2020). In addition, increased serum endocan levels have been observed in patients with rheumatological diseases, such as rheumatoid arthritis and systemic lupus erythematosus (Balta et al., 2013; Balta I. et al., 2014; Icli et al., 2016). These diseases are often associated with systemic inflammation and oxidative stress, which can exacerbate atherosclerosis and increase the risk of cardiovascular diseases. Thus, elevated endocan levels in these patients may serve as a marker of atherosclerosis and indicate a higher cardiovascular risk (Icli et al., 2016; Tuzcu et al., 2019). Moreover, in patients with large-artery atherosclerosis-related ischemic stroke, serum endocan levels were found to be higher than in the control group (P < 0.001); this difference remained significant after adjusting for other risk factors (P < 0.001) (He et al., 2018).

#### 4.1.2 Hypertension

Hypertension is another significant risk factor for ischemic stroke. Clinical data suggest that an increase in endocan levels by 1pg/ml increases the incidence of hypertension by 32.2% (Klisic et al., 2020b). However, the accuracy of these data remains to be verified. It has been shown that the concentration of endocan is low under physiological conditions. However, the average physiological endocan levels measured in different studies varied from a few to hundreds of picograms per milliliter, which may be attributed to the different sensitivities of the enzyme-linked immunosorbent assay kits used and to experimental errors. Moreover, it has been widely confirmed that the median serum endocan concentration in patients with hypertension is significantly higher than that in healthy controls (Balta S. et al., 2014; Musialowska et al., 2018).

In patients with hypertension, endocan was found associated with cIMT, microalbuminuria, and increasing severity of cardiovascular diseases (Balta S. et al., 2014; Wang et al., 2015; Xiong et al., 2015; Oktar et al., 2019). Endothelial activation and dysfunction may also account for this phenomenon. The damaged endothelium releases more free endocan into the bloodstream. Under the action of endocan, circulating lymphocytes adhere and infiltrate the vascular wall, leading to damage of the vascular bed in the structure and thickening of the wall, which further impairs both endothelium-dependent and endothelium-independent vasodilatation. In addition, higher endocan levels also indicate the progression of target-organ damage.

Several kinds of blood pressure drugs have been suggested to lower serum endocan levels in clinical trials. β-blockers, particularly nebivolol and metoprolol, have been discussed in this context (Fici et al., 2013). However, the effects of angiotensin-converting enzyme inhibitors (ACEIs) and angiotensin receptor blockers (ARBs) on serum endocan levels have been less consistently reported. Some studies have observed that antihypertensive treatments can influence serum endocan levels, but these effects are not consistently attributed to specific drug classes like ACEIs and ARBs (de Souza et al., 2016). These mechanisms may involve the vascular protective effects of such medicines and the recovery of endothelial function.

#### 4.1.3 Diabetes mellitus

Diabetes mellitus is strongly associated with an increased risk of ischemic stroke. Several studies have identified a correlation between serum endocan levels and diabetes mellitus (Arman et al., 2016; Lv et al., 2017; Klisic et al., 2020a). Endocan levels were associated with glycated hemoglobin A1 levels and cIMT (Lv et al., 2017; Klisic et al., 2020a). In addition, higher endocan levels might be a reliable marker of diabetes-related complications (Ekiz-Bilir et al., 2019). Importantly, endocan concentration decreases after appropriate antihyperglycemic treatments. In the study by Arman et al. (2016), involving 77 diabetic patients, after three months of antihyperglycemic treatment, patients’ endocan levels decreased from a baseline of 1.55 ± 0.99 ng/mL to 1.07 ± 0.71 ng/mL (Arman et al., 2016).

Vascular endothelial dysfunction is a significant factor in the progression and complications of diabetes. Oxidative stress plays an important role in endothelial injury during hyperglycemia. Mitochondrial dysfunction induced by hyperglycemia also leads to oxidative stress, metabolic dysregulation, and apoptosis in vascular endothelial cells (Sun et al., 2022). In patients with diabetes, the glycocalyx distribution covering the vascular endothelium, which participates in several important vascular functions, is decreased (Dogneì et al., 2018). Zolali et al. (2019) found a decline in cell viability and migration capacity under high glucose levels, and the synthesis and release of endocan by HUVECs were increased under these conditions, suggesting endothelial activation and injury.

Endothelial dysfunction promotes vascular remodeling as well as inflammatory and thrombotic responses, which induce various macrovascular and microvascular complications (Klisic et al., 2023). Moreover, the systemic inflammatory responses induced by hyperglycemia damage the vascular endothelium and induce the expression of several inflammatory cytokines, which, in turn, increases serum endocan levels. A higher glucose concentration (30 mmol/L) may induce a decreased ability of cells to store endocan within cytoplasm, which further increases extracellular endocan levels (Zolali et al., 2019). Endocan can also promote endothelial dysfunction as a mediator of systemic inflammation and oxidative stress. Thus, the vicious cycle involving endocan accelerates disease progression, inducing multi-organ dysfunction.

#### 4.1.4 Other vascular risk factors

Obesity and metabolic syndrome are independent risk factors for ischemic stroke. The relationship between endocan levels and obesity remains controversial. Several experts have suggested that patients with obesity or overweight or patients with metabolic syndrome have lower serum endocan levels than do healthy controls (Janke et al., 2006; Boyuk et al., 2020). Janke et al. hypothesized that the adipose tissue of obese people produced less endocan than that of healthy subjects, which decreased the serum endocan levels (Janke et al., 2006). However, other studies have obtained contradictory results (Arman et al., 2016; Nalbantoğlu et al., 2021). The Body Adiposity Index (BAI) and Cardiometabolic Index (CMI) are two novel anthropometric indices used for rough estimation of the total body fat and visceral adipose tissue distribution, respectively. A previous study (Klisicì et al., 2021) demonstrated that both the BAI and CMI were independently correlated with higher serum endocan levels in adults, with a 12% or 260% increase of a higher endocan level for every 1 unit rise in the BAI or CMI, respectively. Increased endocan levels may reflect endothelial activation in subjects with obesity, which may promote atherogenesis as a proatherogenic inflammatory marker (Nalbantoğlu et al., 2021).

In the circulatory system, the endothelium covering the vessel surface is involved in various essential physiological processes. Smoking, obstructive sleep apnea, hyperhomocysteinemia, and dyslipidemia are thought to induce a systemic proinflammatory state that causes persistent vascular endothelial damage. The activated endothelium releases more endocan, which induces further immune responses. Ultimately, impaired vascular function results in ischemic tissue or organ injuries.

Cardioembolism is another common cause of ischemic attack. A prospective study detected increased endocan levels in an asymptomatic population with atrial fibrillation (AF) (PalaÌ et al., 2022). The CHA2DS2-VASc score is extensively used to assess the risk of stroke in patients with AF. The endocan level predicted a high stroke risk (CHA2DS2-VASc ≥ 2) with 82.5% sensitivity and 71.2% specificity, at a cutoff value of 1.342 (Ceyhun, 2021). Nevertheless, further clinical investigations are needed to confirm these finding.

#### 4.1.5 Ischemia-reperfusion injury

Vascular endothelial cells, pericytes, and astrocytes together comprise the blood-brain barrier (BBB), which presents a natural defense against potential biological and chemical damage to brain tissue (Bai et al., 2022). Following transient ischemic hypoxia, reperfusion of blood flow may induce cerebral ischemia-reperfusion injury, which involves a series of pathological processes, including inflammation, oxidative stress, apoptosis, and various modes of cell death.

In patients with stroke, VEGF levels were increased in the ischemic penumbra. Overexpression of VEGF enhances the permeability of the BBB in the acute phase of stroke, which aggravates ischemia-reperfusion injury. A study (Rocha et al., 2014) using ESM1 -knockout mice found that

380 endocan regulated the extravasation of leukocytes, and ischemic stroke mice lacking *ESM-1* shown a 50% decrease in cerebral edema related to VEGF-induced vascular permeability. In the chronic stage of ischemix xc injury, VEGF plays role in mediating angiogenesis, neuroprotection, and synaptic function (Moon et al., 2021; Hu et al., 2022). HIF is a transcription factor that can directly initiate and activate the transcription of VEGF, which further increases endocan expression. It has been suggested that inhibition of the HIF-1α/ VEGF pathway could significantly reduce ischemia-reperfusion injury to BBB (Hu et al., 2022). As stated earlier, endocan can facilitate binding between VEGF and its receptors (Rocha et al., 2014). Thus, endocan may be a potential target for improving the prognosis of ischemic attacks. In addition, with the disruption of the BBB, circulating immune cells and cytokines penetrate ischemic brain tissue. Erdal et al. (2021) verified a significant correlation between endocan and high-sensitivity C-reactive protein levels in patients diagnosed with silent brain infarction (*r* = 0.196, *P* = 0.16), which may illustrate the pro-inflammatory roles of endocan in ischemic stroke to some extent.

Increased ICAM-1 mRNA expression has been confirmed in ischemic areas 1-4h after reperfusion in transient middle cerebral artery occlusion animal models (Cheng et al., 2008). Endothelial cells play an essential role in protecting the brain parenchyma by maintaining the integrity of the BBB during ischemic attacks. Activated endothelial cells express ICAM-1, which can interact with LFA-1 and elicit circulating leukocytes to penetrate the BBB (Kim et al., 2014). Activated macrophages in the brain parenchyma produce ROS, which cause nuclear DNA oxidation and lipid peroxidation. Endocan has been suggested to induce the expression of ICAM-1 and promote ROS production in macrophages, which may aggravate inflammatory impairment in brain tissue (Lee W. et al., 2014; Sun H. et al., 2019; Kartik Kumar et al., 2020).

NF-kB, a protein complex that controls target DNA transcription, is a key driver of inflammation in the human body. Activation of the NF-kB signaling pathway can increase the expression of TNF-α, IL-β, IL-6, and ICAM-1, which further aggravate nerve cell damage and tissue edema (Ye et al., 2021). Endocan has been suggested to activate the NF-kB signaling pathway. In addition, ESM1 is also a target of NF-kB: NF-kB stimulates endocan expression under the influence of cytokines such as VEGF and IL-1β (Abid et al., 2006; Scuruchi et al., 2021). However, whether the interaction between endocan and NF-kB pathway is involved in the cerebral ischemia-reperfusion injury needs to be verified.

### 4.2 Other neurological disorders

Multiple sclerosis (MS), an autoimmune disease of the central nervous system, is characterized by the dysregulation of T cells, macrophages, microglia cells, and several inflammatory cytokines. A previous study found that endocan levels predicted relapse in relapsing-remitting MS (RRMS) with 67% sensitivity and 66.9% specificity (Akil et al., 2021). A possible hypothesis is that a reactivating immune reaction results in disease recurrence, which stimulates an increase in endocan levels.

Similar mechanisms of endothelial dysfunction and inflammation may also be observed in other neurological disorders such as restless leg syndrome (RLS) and PD. Interestingly, RLS has been considered as a protective factor against atherosclerosis (Park et al., 2012). Decreased dopamine and cortisol release in humans during night hours explains the circadian pattern of RLS symptoms. Additionally, the risk of atherosclerosis is lower in cases with Parkinson’s disease (PD). It is likely that dopamine deficiency in the central nervous system contributes to reduced predisposition to atherosclerosis in RLS and PD. However, this hypothesis needs to be thoroughly tested. Patients with RLS had lower endocan levels (212.18 ± 43.59 ng/mL) than controls (387.91 ± 340.58 ng/mL), which coincided with a negative correlation between RLS and atherosclerosis risk (Celik et al., 2015). Further studies concerning the relationship between endocan and PD are warranted.

Inflammation and angiogenesis are also of interest in terms of mechanisms underlying epileptogenesis (Benini et al., 2016; Rana and Musto, 2018). Peripheral and central inflammation damages the BBB, which induces leukocyte infiltration and inflammatory mediator storms. Ultimately, morphological synaptic changes can lead to epilepsy. It has been suggested that the mRNA expression of cytokines IL-1β, IL-6, TNF-α, and VEGF is increased in the hippocampus after seizures (Rana and Musto, 2018). Benini et al. discovered that disturbing angiogenesis by inhibiting VEGFR phosphorylation could prevent the development of hippocampal atrophy and chronic seizures in rats (Benini et al., 2016). A small study (Faruk Ozdemir et al., 2020) with 21 samples found that serum endocan levels in patients with epilepsy were significantly higher than those in the controls. In addition, these increased endocan values recovered to the levels in healthy subjects after surgery. However, this study found a negative correlation between CRP levels and serum endocan levels in these patients. Consequently, the author suggested that endocan may play a more important role in angiogenesis than in inflammation during epileptogenesis (Faruk Ozdemir et al., 2020).

In addition, it has been demonstrated that patients with diabetic peripheral neuropathy have higher serum endocan concentrations than do other patients with diabetes (Bilir et al., 2016). This difference may be ascribed to the progression of diabetes mellitus. However, whether nerve damage produces this difference needs to be clarified.

Vascular disorders and inflammation are the most common causes of central nervous system diseases, and have a simultaneous influence on their development. Endocan is not only a biomarker of vascular endothelial injury, but is also an important mediator of inflammatory reactions. Identification of the link between endocan and neurological diseases, and interventions targeting endocan might have great clinical benefits.

## 5 Current interventions targeting endocan

Several medications have been suggested to act on endocan molecule, such as targeting of endocan release or expression (statins, inhibition of proinflammatory cytokines), directly neutralizing endocan (mAb), or targeting intracellular signaling pathways associated with endocan (inhibition of VEGF, HGF, NF-kB) (Reikvam et al., 2022).

As a relatively specific inducer of endocan expression, the VEGF pathway is among the top candidates for a large-scale mechanistic study. In in vivo and in vitro models, it has been indicated that both VEGFR2-blocking antibody and the multi-targeting receptor tyrosine kinase inhibitor sunitinib could decrease the secretion of endocan (Leroy et al., 2010; Roudnicky et al., 2013). Scherpereel et al. (2003) found that intrathecal injection of the MEP 08 mAb, a specific mAb that recognizes the phenylalanine-rich region of endocan, could delay tumor appearance in vivo by acting against endocan-dependent tumorigenesis. In addition, Yang et al. (2020) demonstrated that a synthetic peptide corresponding to residues 1-27 of endocan, which is part of the N-terminal region critical for endocan’s interaction with EGFR, may reduce lung tumor growth by blocking the binding of endocan to the extracellular domain of EGFR. In addition, because of the importance of the polysaccharide chain of endocan, deglycosylation by chondroitinase might be a potential method to block the endocan pathway. In addition, as an endocan metabolite, the P14 endocan fragment deserves further attention in future studies, as it may exert biological roles different from those of endocan and may play a role as an endocan inhibitor or modulator.

## 6 Discussion

Endocan is a novel soluble dermatostatin proteoglycan (DSPG) expressed predominantly in vascular endothelial cells and recognized for its role in growth promotion, angiogenesis, and inflammatory responses. While its role in cancer and vascular disease has been studied in detail, the potential role of Endocan in neurological disorders is emerging as an important area of research.

### 6.1 Neuroinflammation

Neuroinflammation is a hallmark of several neurological disorders, including multiple sclerosis (MS), Alzheimer’s disease (AD), and Parkinson’s disease (PD). The role of Endocan in neuroinflammation can be specifically understood through its interaction with endothelial cells and the blood-brain barrier (BBB). Endocan may affect the integrity of the BBB, which is critical for neuroinflammatory conditions. Disruption of the BBB permits the entry of immune cells into the central nervous system (CNS), exacerbating inflammation. Specifically, Endocan’s modulation of cell adhesion molecules (CAMs) such as ICAM-1 and VCAM-1 promotes leukocyte passage across the BBB, thereby exacerbating the inflammatory response. This interaction highlights a potential target for therapeutic intervention in diseases where BBB integrity is compromised.

### 6.2 Neurodegenerative diseases

In neurodegenerative diseases such as Alzheimer’s disease and Parkinson’s disease, Endocan’s role is not limited to its inflammatory properties. One area that needs to be studied in depth is how Endocan affects neurovascular health and neuronal survival. In Alzheimer’s disease, where neurovascular dysfunction is a key early event, Endocan affects angiogenesis by interacting with VEGF and may also affect cerebral blood flow and oxygenation, which are critical for neuronal health. In addition, Endocan’s interaction with signaling pathways such as Wnt/β-catenin and NF-κB may directly affect neuronal survival and apoptosis. Understanding these mechanisms may open new avenues for protecting neurons in neurodegenerative diseases.

### 6.3 Cerebrovascular health

The role of Endocan in cerebrovascular health is multifaceted. It is not only involved in endothelial dysfunction, but also plays an important role in the pathophysiology of stroke and other cerebrovascular diseases. For example, during ischemic stroke, Endocan levels are elevated and correlate with the severity of endothelial damage and inflammation. In addition to its diagnostic potential, targeting Endocan may help to mitigate ischemic damage, preserve endothelial function, and reduce inflammatory damage. Further study of Endocan expression dynamics in the acute and recovery phases of stroke could provide insights into its role in vascular repair and neuroprotection.

### 6.4 Therapeutic implications

Given the multiple roles of Endocan in neurological disorders, therapeutic strategies targeting Endocan may hold promise. Potential interventions include the development of specific antibodies or small molecule drugs to inhibit the pro-inflammatory and pro-angiogenic activities of Endocan. In addition, modulation of Endocan expression or its downstream signaling pathways (e.g., NF-κB and VEGF) may provide neuroprotection. Clinical trials targeting these approaches could help determine the efficacy and safety of Endocan-targeted therapies in the treatment of neuroinflammatory and neurodegenerative diseases.

While a great deal is now known about the role of Endocan in cancer and vascular disease, its importance in neurological disorders is an exciting frontier. By exploring the complex mechanisms of Endocan in the CNS, we can better understand its potential as a biomarker and therapeutic target for neuroinflammation, neurodegenerative diseases, and cerebrovascular health.
